# The Good and the Bad: Ecological Interaction Measurements Between the Urinary Microbiota and Uropathogens

**DOI:** 10.3389/fmicb.2021.659450

**Published:** 2021-05-10

**Authors:** Laurens E. Zandbergen, Thomas Halverson, Jolanda K. Brons, Alan J. Wolfe, Marjon G. J. de Vos

**Affiliations:** ^1^Microbial Eco-Evolutionary Medicine Group, Groningen Institute for Evolutionary Life Sciences, University of Groningen, Groningen, Netherlands; ^2^Department of Microbiology and Immunology, Stritch School of Medicine, Health Sciences Division, Loyola University Chicago, Maywood, IL, United States

**Keywords:** microbiology, ecology, uropathogens, bacterial interactions, infection, UPEC

## Abstract

The human body harbors numerous populations of microorganisms in various ecological niches. Some of these microbial niches, such as the human gut and the respiratory system, are well studied. One system that has been understudied is the urinary tract, primarily because it has been considered sterile in the absence of infection. Thanks to modern sequencing and enhanced culture techniques, it is now known that a urinary microbiota exists. The implication is that these species live as communities in the urinary tract, forming microbial ecosystems. However, the interactions between species in such an ecosystem remains unknown. Various studies in different parts of the human body have highlighted the ability of the pre-existing microbiota to alter the course of infection by impacting the pathogenicity of bacteria either directly or indirectly. For the urinary tract, the effect of the resident microbiota on uropathogens and the phenotypic microbial interactions is largely unknown. No studies have yet measured the response of uropathogens to the resident urinary bacteria. In this study, we investigate the interactions between uropathogens, isolated from elderly individuals suffering from UTIs, and bacteria isolated from the urinary tract of asymptomatic individuals using growth measurements in conditioned media. We observed that bacteria isolated from individuals with UTI-like symptoms and bacteria isolated from asymptomatic individuals can affect each other’s growth; for example, bacteria isolated from symptomatic individuals affect the growth of bacteria isolated from asymptomatic individuals more negatively than vice versa. Additionally, we show that Gram-positive bacteria alter the growth characteristics differently compared to Gram-negative bacteria. Our results are an early step in elucidating the role of microbial interactions in urinary microbial ecosystems that harbor both uropathogens and pre-existing microbiota.

## Introduction

The human body harbors numerous populations of microorganisms in various ecological niches (Turnbaugh et al., 2007). Some of these microbial niches, such as the human gut and the respiratory system, are well studied. One system that has been understudied is the urinary tract, primarily because it has been considered sterile in the absence of infection (Thomas-White et al., 2016). Thanks to modern sequencing and enhanced culture techniques, it is now known that a urinary microbiota exists (Dong et al., 2011; Siddiqui et al., 2011; Fouts et al., 2012; Nelson et al., 2012; Pearce et al., 2014; Wolfe et al., 2012; Lewis et al., 2013; Hasman et al., 2014; Hilt et al., 2014; Willner et al., 2014; Karstens et al., 2016; Brubaker and Wolfe, 2017; Gottschick et al., 2017; Shrestha et al., 2018; Thomas-White K. et al., 2018; Barraud et al., 2019). At least 100 different species have been isolated from urine obtained by transurethral catheterization, mostly from women (Thomas-White K. J. et al., 2018). The implication is that these species live as communities in the urinary tract, forming microbial ecosystems (Zhang et al., 2015). However, the interactions between species in such an ecosystem remain unknown.

For many years, Gram-negative bacteria have been the primary focus of urinary tract infection (UTI) studies (Schaberg et al., 1976; Neu, 1992; Rahman et al., 2009; Peleg and Hooper, 2010; Zowawi et al., 2015; Mazzariol et al., 2017) and these bacteria are generally thought to cause over 85–90% of UTIs caused by bacteria (Flores-Mireles et al., 2015; Mambatta et al., 2015). More recently, however, there has been increased awareness of the presence of Gram-positive bacteria in the urinary tract (Croxall et al., 2011; Kline and Lewis, 2017; Thomas-White K. et al., 2018). In fact, bacteria isolated from asymptomatic persons are most often Gram-positive, with the most common belonging to the genera *Lactobacillus*, *Gardnerella*, *Corynebacterium*, *Streptococcus*, and *Staphylococcus* (Hilt et al., 2014; Pearce et al., 2014). The presence of Gram-positive bacteria in the adult female bladder is likely due to the interconnection between the urinary and genital tracts (Thomas-White K. et al., 2018).

Various studies in different parts of the human body have highlighted the ability of the pre-existing microbiota to alter the course of infection by impacting the pathogenicity of bacteria either directly or indirectly (Arndt and Ritts, 1961; Boris and Barbés, 2000; Stecher and Hardt, 2008; Fava and Danese, 2011; Ubeda and Pamer, 2012; Bäumler and Sperandio, 2016). For the urinary tract, the effect of the resident microbiota on uropathogens and phenotypic microbial interactions is largely unknown (Whiteside et al., 2015). No studies have yet measured the response of uropathogens to the resident urinary bacteria.

The uropathogens are thought to originate from the host’s gut, where the local environment is very different from the urinary tract (Ronald, 2002). Bacteria invading the urinary tract therefore need to adapt to the different urinary environment, which is generally rich in nitrogen but poor in other nutrients, such as carbon (Alteri and Mobley, 2015). Bacteria isolated from individuals experiencing a UTI have been shown to affect each other’s growth (de Vos et al., 2017). Such growth-affecting interactions could potentially play a role in the establishment of uropathogenic bacteria in the urinary tract microbial ecosystem, either aiding or inhibiting their growth.

In this study, we investigate the interactions between uropathogens isolated from the urinary tracts of symptomatic individuals and bacteria isolated from the urinary tract of asymptomatic individuals. To achieve this aim, we studied growth in conditioned artificial urine medium, looking for interactions that affect growth rate, population size, and lag phase. Experiments that measure the effect on such growth characteristics under the influence of conditioned media prepared from the cell-free supernatants of other bacteria have been shown to be a good proxy for bacterial interactions (de Vos et al., 2017). We observed that bacteria isolated from individuals with UTI-like symptoms and bacteria isolated from asymptomatic individuals do affect each other’s growth; bacteria isolated from symptomatic individuals affect the growth of bacteria isolated from asymptomatic individuals more negatively than vice versa. Additionally, we show that Gram-positive bacteria alter the population size and the length of the lag phase differently compared to Gram-negative bacteria. Our results are a first step in elucidating the role of microbial interactions in urinary microbial ecosystems that harbor both uropathogens and pre-existing microbiota.

## Materials and Methods

### Bacterial Isolates

Urinary tract infection isolates were obtained anonymously during a previous study from elderly patients (>70 years old) (Croxall et al., 2011); ten were selected based on the results of a previous study (de Vos et al., 2017). Non-pathogenic isolates (commensals) were obtained from asymptomatic women and were previously used in several IRB-approved studies reported in two published papers and two in preparation (Thomas-White K. J. et al., 2018; Price et al., 2020). To verify and obtain single colonies, isolates were diluted by streaking on CHROMagar Orientation (CHROMagar). Identification of bacteria was verified by PCR amplification and sequencing of the 16S rRNA gene (BaseClear; B8F and 1492R primers). Isolates were stored at −80°C in 96-wells plates as glycerol stocks (v/v 25% glycerol). Only those isolates that grew consistently and achieved planktonic growth were used for these experiments. Isolates used during the experiments are listed in Supplementary Table 1.

### Growth Medium

Bacteria were grown in an adapted version of the artificial urine medium (AUM) originally described by Brooks and Keevil (1997). Slight adaptations were made to decrease crystal formation in the medium and to allow for planktonic growth of the isolates. The 1× AUM contained bacto peptone L37 1.5 g/L (BD), NaHCO3 3.15 g/L, urea (Roth) 11.25 g/L, Na2SO4.10H2O 4.8 g/L, K2HPO4 1.8 g/L, NH4Cl 1.95 g/L, bacto yeast extract 15 mg/L (BD), lactic acid (Roth) 1.98 mM, citric acid 600 mg/L, uric acid 105 mg/L, creatinine 1.2 g/L, CaCl2.2H2O 4.44 mg/L, Fe(II)SO4.7H2O 1.8 g/L (Riedel), MgSO4.7H2O 368 mg/L, KH2PO4 1.43 g/L. Chemicals were from Sigma, unless stated otherwise.

### Generation of Conditioned Medium and Replenishment

The bacterial strains were grown in 250 mL 0.8× AUM for 48 h in 500 mL Erlenmeyer flasks at 37°C and shaken at 200 rpm. Cultures were subdivided into 50 mL culture tubes (TPP) and centrifuged at room temperature at 4,800 *g* for 15 min. The supernatants were subsequently filtered using bottle filter tops (TPP), first with a 0.45 μm filter followed by another filtration step with a 0.2 μm filter, resulting in the spent medium from which the conditioned medium was created. The conditioned medium was generated by replenishing the spent media by mixing with fresh AUM medium (without sodium chloride) in such a way that the final concentration in the conditioned medium ranged from 0.6× (for components that were completely consumed in the spent medium) to 1× (for components from which nothing was consumed in the spent medium) of the concentration in fresh AUM. 0.01% (v/v) triton-100 was added for the prevention of biofilm formation and clotting, which have the potential to interfere with OD600 measurements; the medium was buffered with 0.01 M PIPES (pH 6.5).

### Growth Measurements and Conditions

Each isolate was incubated in one well of a prewarmed 96-well plate (transparent non-treated flat bottom, Brand) containing 200 μL of conditioned medium for 24 h. The plates were inoculated using a 96 solid pin multi-blot replicator tool, transferring roughly 1 μL of stock from a −80°C glycerol stock (see bacterial isolates above). Plates were incubated in a Versa max microplate reader at 37°C under aerobic conditions and shaken continuously at 517 rpm. The optical density at 600 nm (OD600) was measured every 6–7 min in the microplate reader (three flashes, 10 ms settle time). Before each measurement, the plate was shaken using the automix feature of the SoftMax^®^ Pro software for 5 s, 517 rpm; double orbital pattern. The growth yield was defined by the maximum OD600 reached and calculated using the first five values of the stationary phase when the bacteria reached this phase. All isolates reached stationary phase within 20 h, with the OD600 largely staying constant during the remaining time. The growth rate was defined by the shortest obtained doubling time and quantified by the steepest linear fit of the exponential phase of log(OD600). Lag phase was defined as the length of time before OD600 reached 0.01. Isolates were measured in triplicate, randomly placed on the 96-well plate, but not in the outside wells. Evaporation of media from the plates was not detected. After measurements, the plates were visually inspected for biofilm formation and isolates that had formed biofilms or clots were discarded from the data. 96-well plates also contained non-inoculated wells as a control for contamination both of the conditioned medium and the inoculation step. Contamination was found to be negligible.

### Analysis of Interactions

The interactions parameter of the population size was calculated using the formula ε = log(Nc/Nu), where Nc is the growth yield in conditioned medium and Nu is the growth yield in unconditioned medium (1× AUM concentration). The growth rate was expressed in fastest doubling time and the interaction parameter was calculated using the formula ε = log(gu/gc). The length of the lag phase was calculated from the amount of time it took after inoculation for the OD600 to reach a value of 0.01. The interaction parameter for the lag phase was calculated using the formula ε = log(Lu/Lc). By calculating the interaction parameters in this manner, a value of ε > 0 would mean the growth in conditioned medium was higher than in the reference unconditioned medium, while a value of ε < 0 would indicate growth smaller than in the reference medium. ε ≈ log(0.6) = −0.51 is the value of the lowest possible level of growth inhibition that can be attributed to resource overlap alone, as it corresponds to the lowest possible concentration of the nutrients in the conditioned medium. Values lower than this indicate that there are other mechanisms that reduce growth, such as production of bacteriocins.

We measured interactions in triplicate in both conditioned and reference medium, and for all measurements there were at least two technical replicates. We did not include donor/acceptor combinations in the statistical analysis if the responses of the acceptor strain deviated more than 10% from the median of the technical and biological replicates (Indicated with a “^∗^” symbol in Supplementary Figure 1). An example of growth curves included and excluded from analysis can be found in Supplementary Figure 2. Interestingly, it seemed that the donors drive this wider spread of interactions, as most acceptors grown in conditioned medium from these specific donors would show larger deviations of the growth parameters. It is possible that these donors create a larger range of metabolites, resulting in a medium with a broader range of options for acceptors to adapt to, or otherwise induce different phenotypes in the population.

### Spot-on-Lawn Assay

For the spot-on-lawn assays, 2× AUM was mixed 1:1 (v/v) with sterile, preheated (∼50°C) agar-demi-water solution (30g/L). 1× AUM agar plates were produced using 20 mL of the AUM-agar mixture. A top layer of agar containing the bacteria to be tested was created by mixing 10 mL of the AUM-agar mixture with 0.25 mL of a stationary phase bacterial culture and pouring it on top of a set 1.5× AUM agar plate. Conditioned media was prepared as described above, and 10 μL drops of the conditioned media were dropped on the agar surface in duplicate after the bacterial top layer was poured and let to set. Plates were then allowed to grow overnight at 37°C and the agar plates were inspected for zones of inhibition, caused by the drops of conditioned media. Fresh 1× AUM was dropped in duplicate on the plate as a negative control. Those bacteria that showed a very negative response ε < log(0.1) = −2 to conditioned liquid media were tested to check for inhibition.

### Statistical Analysis

All experiments were carried out in triplicate to validate reproducibility of the experiments. Welch’s *t*-test was used to calculate *p*-values (two-tailed) for the probability of interactions of one group being equal to that of another tested group, with values of *p* < 0.05 being taken as significantly different. Final growth characteristic comparisons were tested with Bonferroni correction for multiple comparisons (*p* < 0.05).

### DNA Extraction and 16S Analysis

Bacterial strains were streaked on CHROMagar Orientation (CHROMagar) and allowed to grow overnight at 37°C. Bacteria were taken from the agar plate using a sterile inoculation loop and dissolved in AUM. The bacteria were centrifuged at 10,000 × *g* for 2.5 min. Supernatants were discarded and pellets dissolved in 100 μl sterile dH2O which was then used for DNA extraction. DNA extractions were performed using the fast DNA extraction method described by Brons et al. (2020), based on the Qiagen PCR purification kit (Qiagen, Hilden, Germany). Three *Escherichia coli*, two Corynebacterium, and the two *Lactobacillus jensenii* isolates had a too low yield as evidenced from gel electrophoresis (PCR performed using primers B8F and 1492R). New DNA extractions were performed, this time starting from a bacterial culture in AUM, grown overnight at 37°C. Cultures were centrifuged at 10,000 × *g* for 2.5 min. After discarding supernatant, pellets were dissolved in 100 μl dH2O and DNA extraction was performed again using the same method, this time with enough yield for sequencing. The sequencing was performed by BaseClear Inc., (Leiden, Netherlands). The end bases with a quality of <5 were trimmed from the sequences and annotation was performed using Geneious 8.1.3^1^ and the SILVA reference database (Pruesse et al., 2007). Sequences were aligned using the MUSCLE plugin for Geneious (Edgar, 2004). Quality of sequences and absence of chimeras were checked using the SILVA reference database. The phylogenetic tree was constructed using Clustal Omega 1.2.4 (Sievers et al., 2011) software.

## Results

### Bacterial Interactions in Urine-Like Environment

To obtain a basic understanding of the interactions between bacteria isolated from the urinary tracts of symptomatic individuals and those that were asymptomatic, we assessed their interactions in terms of growth characteristics in an *in vitro* growth assay. In this growth assay, we measured the pairwise interactions of uropathogens and commensals using conditioned AUM (section “Materials and Methods”). Interactions on the level of population size, doubling time and length of lag phase were calculated as follows: ε = log(Nc/Nu) for population size, ε = log(gu/gc) for doubling time and ε = log(Lu/Lc) for lag phase (section “Materials and Methods”) (de Vos et al., 2017). Nc is the population size in conditioned medium and Nu in unconditioned medium. The following were considered positive interactions (ε > 0): increased population size, decreased doubling time, and a shorter lag phase. Bacteria grown in conditioned medium are considered acceptors, whereas bacteria from which the conditioned media were generated are considered donors. For simplicity, we will refer to the bacteria isolated from the urinary tracts of asymptomatic women as commensals, and bacteria isolated from persons diagnosed with UTIs as uropathogens, even though we are aware that these relationships can be more complex.

**FIGURE 1:**
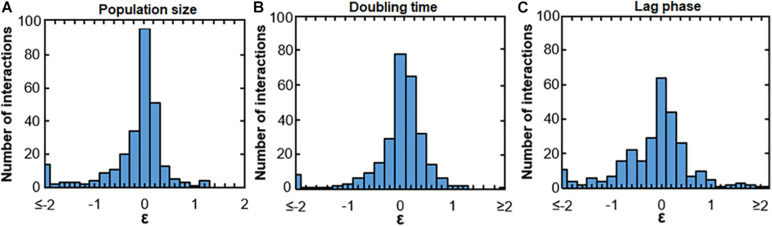
Bacterial interactions of all measured uropathogens and commensals. Histograms show the response of uropathogens and commensals to conditioned medium. Many neutral interactions were observed (value on *x*-axis around 0), Values of ε < log(0.125) = –2 or ε > log(8) = 2 are binned at –2 and 2, respectively. **(A)** Growth interactions affecting population size. Values of ε > log(1) = 0 indicatea larger population size in comparison to the unconditioned reference medium and values of ε < log(1) = 0 indicate a smaller population size. **(B)** Growth interactions affecting the growth rate. Values of ε > log(1) = 0 show an increased maximum growth rate in comparison to the reference medium and ε < log(1) = 0 show a decreased growth rate. **(C)** Growth interactions affecting the length of the lag phase. A value of ε > log(1) = 0 indicates a shorter lag phase and ε < log(1) = 0 indicates a longer lag phase in comparison to growth in the reference medium.

We observed both positive and negative interactions between uropathogens and commensals. Figure 1 shows these interactions in terms of population size, doubling time and lag phase with medians of −0.12, −0.040, and −0.11, respectively, indicating a skewed distribution toward negative interactions (Figures 1A–C). This indicates that negative interactions were observed more often than positive interactions. Most interactions were neutral-to-weak [log(0.8) = −0.22 > ε > log(1.2) = 0.18]. In addition, 38, 51, and 28% of the negative interactions affecting population size, growth rate and lag phase were relatively weak as well [log(0.8) = −0.22 > ε > log(1) = 0]. However, the majority of the negative interactions were rather strong [ε > log(0.6) = −0.51]. Only 11% of the interactions affecting doubling time and 12% of interactions affecting lag phase were positive [ε > log(1.4) = 0.33]. For a very small fraction of interactions, the doubling time and length of the lag phase were more than halved (ε > log(2) = 0.69, 6.4, and 2.9%, respectively). These results indicate that the measured urinary bacteria were more likely to hinder the growth of other urinary bacteria than to stimulate growth. Moreover, the many weak interactions of urinary bacteria were neutral or weakly positive or negative. According to ecological theory, the presence of weak interactions promotes the ecological co-existence in communities (Pfennig et al., 2006; Mougi and Kondoh, 2012; Friedman and Gore, 2017; Kehe et al., 2020).

We expected to find a correlation between interactions that affect the length of lag phase and interactions that affect the growth rate, as a shorter lag phase could indicate that bacteria can reach a certain population size threshold sooner given the available nutrients. A shorter lag phase could potentially allow bacteria to establish themselves faster in the new environment. Trade-offs between population size and growth rate are also well described, either sacrificing growth rate to obtain a larger population size or using a low-yield pathway to increase the growth rate (Novak et al., 2006; Bachmann et al., 2013). This is relevant as the success of infection by pathogens is often density-dependent (Castillo-Juárez et al., 2015). However, in most studies, the correlation between these parameters is rather poor (Velicer and Lenski, 1999; Novak et al., 2006; Maharjan et al., 2007; Fitzsimmons et al., 2010). In this study, interactions affecting population size, doubling time and lag phase were not correlated to each other in any way (Supplementary Figure 3); a shorter doubling time did not correlate with increased or decreased population size, nor did reduced doubling time indicate a longer lag phase. This suggests that bacterial interactions in a urinary-like environment have independent effects on growth rate, population size and lag phase.

Responses were not always species- or genus-specific (Supplementary Figures 1, 2). Even though the responses of different strains of the same species or genus often showed a similar response, this was not always the case. For example, three isolates from the commensal species *Staphylococcus aureus* and *Micrococcus luteus* were tested against conditioned medium of the uropathogen donor *Pseudomonas fluorescens*. The population size and doubling time of one particular commensal isolate of *S. aureus* and one isolate of *M. luteus* were very negatively affected [ε < log(0.125) = −2] (Supplementary Figures 1A,C). Yet, the other isolates of each of these same species exhibited significantly weaker responses to the same donor. This suggests that particular genetic components of a particular strain can be the driver for those specific interactions.

### Different Interactions of Gram-Positive and Gram-Negative Bacteria

**FIGURE 2:**
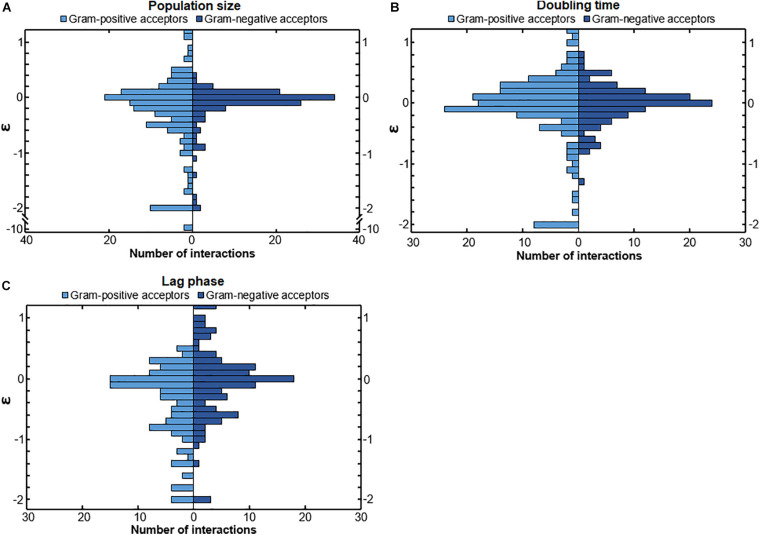
The response of Gram-positive and Gram-negative acceptors. **(A)** Gram-negative bacteria showed a more conserved response compared to Gram-positive bacteria to conditioned media. The distribution of population size interactions was significantly different for Gram-positive bacteria and Gram-negative bacteria (*p* = 0.024), meaning that Gram-positive bacteria overall had a wider range of responses to the conditioned media of the donors **(B)** The distribution of the doubling time interactions of Gram-positive and Gram-negative acceptors was not significantly different (*p* = 0.051), even though Gram-negative bacteria showed a more conserved response with the data points more around the median compared to Gram-positive acceptors. **(C)** The lag-phase of Gram-negative acceptors was more often positively affected (meaning a shortened lag-phase) compared to Gram-positive acceptors. The distribution of interactions affecting the length of the lag phase was significantly different for Gram-positive and Gram-negative bacteria (*p* = 0.0013). Significance of results was calculated using Welch’s unequal variances *t*-test.

We investigated whether Gram-positive and Gram-negative bacteria react differently to other bacteria. We observed a difference in the responses of Gram-positive and Gram-negative bacteria (Figure 2). The population size (Figure 2A) and lag phase (Figure 2C) of Gram-positive and Gram-negative acceptors were affected differently. The distributions of interactions affecting these parameters were significantly different for these two groups (*p* = 0.024 and *p* = 0.0013, respectively). The doubling time (Figure 2B) of Gram-positive and Gram-negative bacteria was also affected differently but did not differ as much as the population size and lag phase (*p* = 0.051). The population size and the doubling time interactions of Gram-positive acceptors showed a wider spread than those of Gram-negative acceptors (Figures 2A,B). Gram-negative acceptors were more likely to experience a reduced lag time and show a wider spread of positive interactions compared to Gram-positive acceptors (Figure 2C). This suggests that Gram-negative bacteria are better adapted to thrive in a urine environment when other bacteria are present.

**FIGURE 3:**
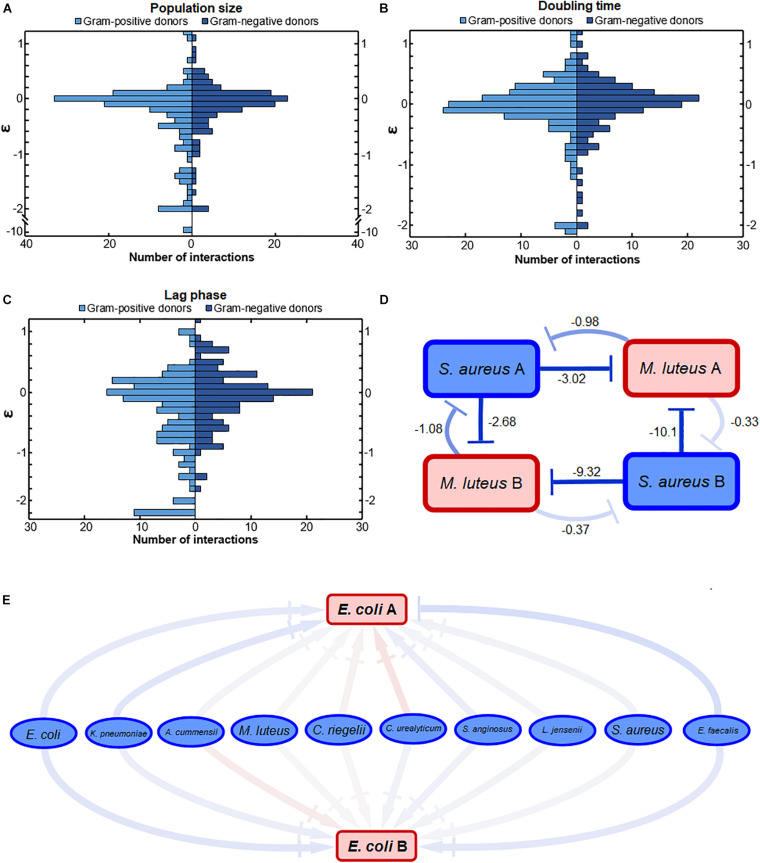
The effect of conditioned media from Gram-positive and Gram-negative donors. **(A)** The effect on population size was significantly different. Conditioned media of Gram-positive donors led to a wider range of growth effects compared to the effect of Gram-negative donors (*p* = 0.032). Mostly, the Gram-positive donors more often had a strong negative effect on population size compared to the Gram-negative donors. **(B)** Gram-positive and Gram-negative donors did not have a significantly different effect on the doubling time interactions. The distributions of interactions between Gram-positive and Gram-negative donors was relatively similar (*p* = 0.17). **(C)** Gram-negative donors induced a more conserved response on the lag phase compared to Gram-positive donors. This could suggest that the Gram-negative bacteria produce a more similar environment compared to Gram-positive bacteria. The distribution of interactions affecting the lag phase of acceptors by Gram-positive and Gram-negative donors was significantly different (*p* = 0.0014). **(D)** Strong negative bacterial interactions in conditioned media. Two pathogenic *S. aureus* donors inhibited the population size of two commensal *M. luteus* isolates strongly. The numbers next to the connectors show the ε-values of the interactions. The darker the color of the connector, the stronger the interaction. Red indicates positive, whereas blue indicates negative interactions. **(E)** Weak interaction effects of commensal donor conditioned media on the population size of two pathogenic *E. coli* isolates. Color gradient of the connectors indicate interactions strength, with |→ connectors indicating a weak-to-neutral interaction [log(0.8) = –0.22 < ε < log(1.2) = 0.18]. The darker the color of the connector, the stronger the interaction. Red indicates positive, whereas blue indicates negative interactions. Significance of results was calculated using Welch’s unequal variances *t*-test.

We also investigated whether Gram-positive or Gram-negative bacteria affect other bacteria differently. We observed a difference in the response to either Gram-positive or Gram-negative donors (Figure 3). Gram-positive and Gram-negative donors differentially affected the population size (Figure 3A) and lag phase (Figure 3C), as can be observed from the different interaction distributions (*p* = 0.032 and *p* = 0.0014, respectively). In contrast, Gram-positive and Gram-negative donors did not affect the doubling time (Figure 3B) differently, as there was no significant difference between the interaction distributions (*p* = 0.17).

In general, the response to Gram-positive bacteria was more diverse than the response to Gram-negative bacteria. These response differences correlated with the phylogenetic distance between the bacteria, where the Gram-positive bacteria were more distant from each other than the Gram-negative bacteria (Supplementary Figure 4). Gram-positive bacteria, for instance, tended to cause either strong positive or negative lag phase interactions (Figure 3C). Gram-positive donors also induced a stronger negative response on the population size (Figure 3A). In general, the more phylogenetically diverse Gram-positive donors tended to induce a more varied response than the Gram-negative donors (Supplementary Figure 4).

An illustration of the nature of a few strong interactions can be found in Figure 3D. Two pathogenic *S. aureus* isolates have strong negative effect on the population size of two commensal *M. luteus* isolates. Interestingly, the *S. aureus* isolate exhibiting the strongest negative interactions (ε = 10.1 and ε = −9.32) was least affected by both *M. luteus* isolates.

An illustration of a few weak interactions is described by two pathogenic *E. coli* isolates (Figure 3E). Their population size was hardly affected by any of the commensal isolates. This can potentially be caused by low niche overlap due to the metabolic flexibility of uropathogenic *E. coli* (Alteri et al., 2019).

**FIGURE 4:**
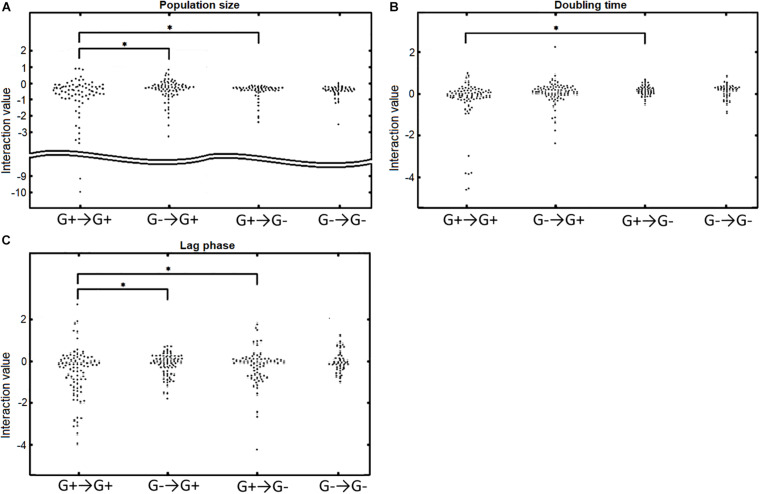
The response of Gram-positive (G+) and Gram-negative (G–) acceptors to Gram-positive or Gram-negative donors. G+→G+ indicates the response of Gram-positive acceptors to Gram-positive donors, G–→G+ indicates the response of Gram-positive acceptors to Gram-negative donors, G+→G– indicates the response of Gram-negative acceptors to Gram-positive donors, and G–→G– indicates the response of Gram-negative acceptors to Gram-negative donors. **(A)** Population size interactions.Gram-positive acceptors responded significantly different to Gram-positive and Gram-negative donors (*p* = 0.031). In contrast, Gram-negative acceptors did not respond differently to Gram-positive or Gram-negative donors (*p* = 0.70). Gram-positive and Gram-negative acceptors responded significantly different to Gram-positive (*p* = 0.022), but not to Gram-negative donors (*p* = 0.63). **(B)** The effect of interactions on doubling time. There was a significant difference for Gram-positive and Gram-negative acceptors responding to Gram-positive donors (*p* = 0.023), but not to Gram-negative donors (*p* = 0.90). No significant difference was observed for Gram-negative acceptors responding to either Gram-positive donors or Gram-negative donors (*p* = 0.076). Neither was there a difference in doubling time of Gram-positive acceptors responding to Gram-positive or Gram-negative donors (*p* = 0.30). **(C)** Effect of interactions on the acceptors’ length of the lag phase. Gram-positive acceptors respond significantly different to Gram-positive and Gram-negative donors (*p* = 0.0031). Such a difference was not observed for Gram-negative acceptors (*p* = 0.093). Gram-positive and Gram-negative acceptors also responded significantly different to Gram-positive donors (*p* = 0.024). Significance of results was calculated using Welch’s unequal variances *t*-test.

Since we observed a general difference in the response to Gram-positive bacteria and Gram-negative bacteria, we wondered whether Gram-positive and Gram-negative bacteria also react differently from each other to the different donors (Figure 4). We found that Gram-positive acceptors react differently to Gram-positive and Gram-negative donors. Particularly, the population size (Figure 4A) and the length of the lag phase (Figure 4C) were affected differently by Gram-positive or Gram-negative donors, as can be observed by the significant difference in the distribution (*p* = 0.031 and *p* = 0.0031, respectively). In contrast, Gram-negative bacteria did not react differently to Gram-positive and Gram-negative donors; there was no significant difference in the interaction distributions for all three growth parameters (*p* = 0.70, *p* = 0.90, *p* = 0.093 for population size, doubling time, and length of lag phase, respectively).

We also observed that Gram-positive and Gram-negative acceptors react differently to Gram-positive donors. The interactions distributions for population size, doubling time and lag phase (*p* = 0.022, *p* = 0.023, and *p* = 0.024, respectively) were different for the Gram-positive and Gram-negative bacteria reacting to Gram-positive donors (Figure 4). These differences were not observed for the response to Gram-negative donors. As mentioned before, this is correlated with the larger genetic diversity of Gram-positive bacteria compared to Gram-negative bacteria, with a larger phylogenetic distance being linked to larger growth changes (*p* = 0.041, *p* = 0.018, and *p* = 0.038 for population size, doubling time and lag phase, respectively) (Supplementary Figure 4). The results also show that Gram-positive bacteria are more likely to affect the growth of a bacterium very negatively if the interaction is negative, suggesting a potential role for Gram-positive bacteria in protecting the human urinary tract against potential pathogenic invaders.

### UTI Pathogens Versus Commensals

**FIGURE 5:**
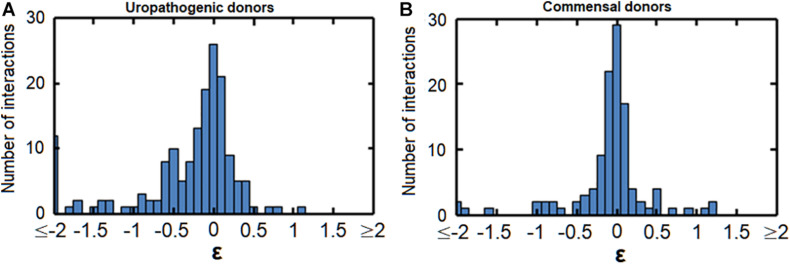
Histograms of the interactions between commensals and uropathogens. **(A)** The effect of uropathogenic donors on the population size of commensal acceptors. **(B)** The effect of commensal donors on the population size of uropathogenic acceptors. Commensal acceptors exhibited a more negative response (<ε = log(0.6) = –0.51) to uropathogens than uropathogens to commensals. A similar pattern was observed for interactions affecting the doubling time and the length of the lag phase (Supplementary Figure 5).

**FIGURE 6:**
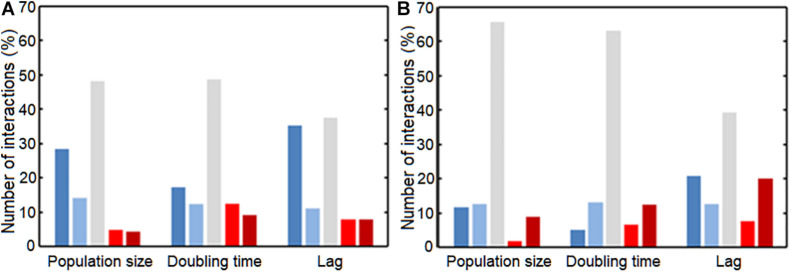
Interactions between commensals and pathogens, classified according to the interaction strength. Interaction strength was subdivided in very negative (Dark blue), slightly negative (Light blue), neutral (Gray), slightly positive (Light red), and very positive (Dark red). Groups were divided based on ε-value; very negative: ε < log(0.6) = –0.51, slightly negative: log(0.6) = –0.51 < ε < log(0.8) = –0.22, neutral: log(0.8) = –0.22 < ε < log(1.2) = 0.18, slightly positive: log(1.2) = 0.18 < ε < log(1.4) = 0.33, and very positive: ε > log(1.4) = 0.33 interactions. **(A)** Commensal acceptors were exposed to conditioned medium of uropathogen donors. **(B)** Uropathogen acceptors were exposed to conditioned medium of commensal donors. For the very negatively affected interactions, there was a significant difference for the affected population size, doubling time and lag phase between the commensals and uropathogens (*p* = 0.0036, *p* = 0.0042, *p* = 0.0144, respectively). Uropathogens showed a significant shortening of the lag phase compared to commensals (*p* = 0.0072). Significance of interaction comparisons was tested using a *t-*test with Bonferroni correction for multiple comparisons (*p* < 0.05).

**FIGURE 7:**
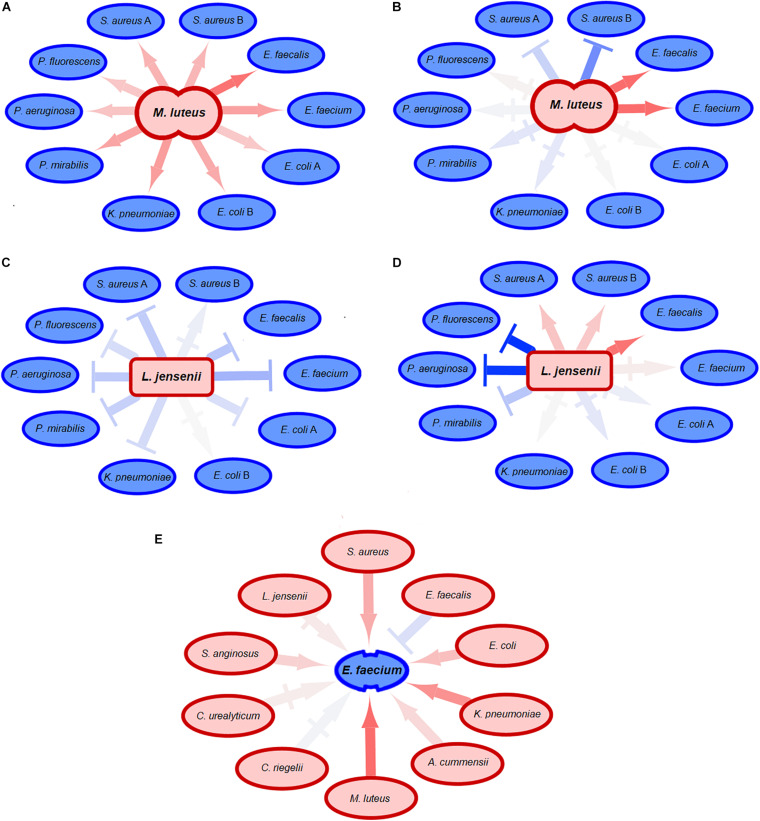
Effect of commensal acceptors on pathogens. Neutral-to-weak interactions are symbolized by |→ connectors. The darker the color of a connector, the stronger the growth effect of the donor was. Red connectors indicate positive interactions, blue connectors negative interactions. **(A)** The conditioned medium of the commensal donor *M. luteus* shortens the lag phase of many uropathogens. The effect of commensal donors on uropathogens seems to be donor-driven. **(B)** The conditioned medium of the same commensal *M. luteus* donor as in **(A)** does not have comparable effects on the population size of the tested uropathogenic acceptors, indicating that the effect on population size of acceptors is not donor-driven. **(C)** The commensal donor *L. jensenii* increases the length of the lag phase of uropathogenic acceptors. Just like in **(A)**, effects on the lag phase of uropathogens by commensals suggests that the effect on lag-phase is donor-specific. **(D)** *L. jensenii* does not have comparable effects on the population size of uropathogens, suggesting that the effect of commensals on uropathogenic acceptors is not donor-driven. **(E)** The population size of uropathogenic *E. faecium* is positively affected by commensal donors, both the Gram-positive and Gram-negative donors with the exception of another *Enterococcus*. This suggests that the effect on population size of *E. faecium* is not donor-specific, but the population size is positively affected by the presence of any commensal in comparison to isolated growth except for closely related species.

We next asked whether uropathogenic acceptors react differently to commensal donors than vice versa (Figure 5 and Supplementary Figure 5). There was a clear difference between uropathogens and commensals, with more very strong negative interactions [ε < log(0.6) = −0.51] caused by the uropathogenic donors compared to commensal donors (*p* = 0.0036, *p* = 0.0042, and *p* = 0.014 for population size, doubling time and length of lag phase response) (Figure 6). Interestingly, most strongly negative interactions ε < log(0.22) = −1.5 were caused by uropathogenic donors. This suggests that uropathogens are better competitors, or that the uropathogens on average have a broader niche. Interestingly, in some cases closely related donors caused this very negative interaction (Supplementary Figure 4). This is indicative of some form of niche control by bacteria (e.g., bacteriocins) to improve the odds of survival in an environment that includes competitors (Riley and Wertz, 2002; Kommineni et al., 2015). However, the effect of inhibiting compounds could not be detected using spot-on-lawn assays (data not shown). The only parameter for which there was a significant difference for positive interactions between uropathogens and commensals [ε > log(1.6) = −0.51] was the length of the lag phase (*p* = 0.0072, Figure 6); uropathogens had a more reduced lag time induced by commensal donors compared to commensal bacteria under the influence of uropathogenic donors. The positive effect on the lag phase seems to be driven by the conditioned medium of the commensal donor. An example of this is the conditioned medium of the commensal *M. luteus* isolates (Figure 7A). Interestingly, this donor-driven effect is not observed for the effect on population size and doubling time (Figure 7B). The donor-driven effect of the commensal conditioned media on the lag phase is also observed for the negative and neutral interactions, like those of the commensal *L. jensenii* (Figure 7C). Again, this was not observed for the population size and doubling time (Figure 7D). This indicates that the effect of the established isolate or community on the lag-phase of uropathogenic bacteria is not pathogen-specific, but rather community-specific. The population size, and to a lesser extent the doubling time (Supplementary Figure 1D), of pathogenic *Enterococcus faecium* was mostly positively affected in the conditioned media of the tested commensals (Figure 7E), which is in line with what has been observed previously (de Vos et al., 2017). This would suggest that pathogenic *E. faecium* benefits from the (past) presence of other organisms in the environment.

## Discussion

We obtained insight into interactions between bacteria isolated from the urinary tracts of women with no lower urinary tract symptoms (commensals) and bacteria isolated from elderly patients diagnosed with UTIs (uropathogens). There are indications that commensal bacteria can play a role in the growth and establishment of uropathogens (Nienhouse et al., 2014; Neugent et al., 2020), and our research is an early step in elucidating the potential interactions of the urinary microbiota in a urinary environment. An increased understanding of the microbiota and the ecology of the human urinary tract may create opportunities for the development of treatment strategies based on ecological principles (Hibbing et al., 2010), harvesting the knowledge of specific ecological interactions of the urinary microbiota and uropathogens.

For simplicity, we have made the clear distinction between commensals isolated from asymptomatic women and uropathogens isolated from patients diagnosed with UTI. We do not know the gender of the elderly patients from whom the pathogens were isolated, thus we cannot draw conclusions based on gender differences in UTI that are known to exist (Harrington and Hooton, 2000; Brubaker et al., 2021). We also realize that the host may play a large role in the potential pathogenicity of uropathogens (Finlay and Falkow, 1997; Nielubowicz and Mobley, 2010). Yet, it is intriguing to think of ecological interactions between other bacteria as inducers or quenchers of pathogenicity, as it is known that density-dependent molecular mechanisms play a role in infections (Castillo-Juárez et al., 2015). Together with previous results, our results show that many interactions of urinary bacteria are neutral or weakly positive or negative. According to ecological theory, the presence of weak interactions promotes the ecological co-existence in communities (Pfennig et al., 2006; Mougi and Kondoh, 2012; de Vos et al., 2017; Friedman and Gore, 2017; Kehe et al., 2020).

We found that Gram-positive donors led to larger negative growth effects in other bacteria, perhaps suggesting that these Gram-positive bacteria are more likely to produce interfering metabolites. Those strains that had a strong negative effect on uropathogens were quite often closely related species or even different isolates from the same species. We also found that commensals are more negatively affected by uropathogens than vice versa. These results could be an indication that (1) there are smaller environmental challenges for the uropathogens in conditioned media created from commensal donors, (2) there is less niche overlap or uropathogens have a larger niche, (3) uropathogens are more metabolically flexible and thus can change their metabolism to environmental demands, or (4) competitive metabolites produced by the commensals have a smaller effect on uropathogens (Swinnen et al., 2004).

Our experimental design allowed for high-throughput measurements of pairwise interactions, but such an approach cannot encompass the complexity of the urinary environment. Even though bacterial communities in the urinary tract are considerably less rich when compared to communities in other niches (e.g., the human gut), interactions between the host and the multiple species that the host harbors could have different effects on those species (Hilt et al., 2014; Trosvik and de Muinck, 2015; Brubaker and Wolfe, 2017; Momeni et al., 2017). Our conditioned medium assay cannot account for such higher-order bacterial interactions, *in vivo* metabolic effects, and direct or physical reciprocal interactions (Cornforth and Foster, 2013; Kelsic et al., 2015). Also, interactions with the host, especially host-pathogen interactions, can have a marked effect on community structure since uropathogens are known to attach to and invade the bladder epithelial cells (Kim et al., 2008; Lemonnier et al., 2008; Kline et al., 2009; Nielubowicz and Mobley, 2010; Horsley et al., 2013; Flores-Mireles et al., 2015). Interactions can also be dependent on different environments, and urine composition can be affected by various elements, both within and between individuals (Bouatra et al., 2013). Yet, it was previously found that pairwise interactions could, for the larger part, predict the dynamics of larger populations (Guo and Boedicker, 2016; de Vos et al., 2017). Future studies are required to include all factors that can affect population dynamics to obtain full understanding of the urinary microbiota, which will require *in vivo* models to ecologically define the characteristics of these communities. This will in turn lead to a better understanding of the urinary pathobiome (Vayssier-Taussat et al., 2014), and whether colonization of a pathogen leads to infection is dependent on the ecological background and the commensal microbiota (Andersen et al., 2019; Bass et al., 2019).

Shedding light on the underlying molecular mechanisms also will help to better understand community dynamics and facilitate development of treatment strategies. To conclude, this work is an early step toward understanding urinary community dynamics, and we have shown that urinary commensals should not be ignored as they can have effect on the growth of uropathogenic bacteria in a urine-like environment.

## Data Availability Statement

The data presented in the study are deposited in the GenBank repository under accession numbers MW565925-MW565973 and MW890540.

## Author Contributions

LZ and MV designed the experimental plans and analyzed the data. LZ, JB, and TH performed the experiments. LZ, MV, and AW prepared the manuscript. All authors contributed to the article and approved the submitted version.

## Conflict of Interest

The authors declare that the research was conducted in the absence of any commercial or financial relationships that could be construed as a potential conflict of interest.

## Footnotes

1 https://www.geneious.com
