# Supplementary material for: The Good and the Bad: Ecological Interaction Measurements Between the Urinary Microbiota and Uropathogens
Source: Front Microbiol. 2021 May 10;12:659450. doi: 10.3389/fmicb.2021.659450 (PMC8141646; doi:10.3389/fmicb.2021.659450)
Supplement: Supplementary file 1 [file Data_Sheet_1.docx]

**Table S1. Bacterial isolates, commensals and uropathogens.** Uropathogens are bacteria isolated from patients with an UTI and commensals are bacteria not associated with UTI.

| **Commensals** | **# of isolates** | **Uropathogens** | **# of isolates** |
| --- | --- | --- | --- |
| *Arthrobacter cumminsii* | 1 | *Escherichia coli* | 2 |
| *Corynebacterium urealyticum* | 1 | *Enterococcus faecalis* | 1 |
| *Corynebacterium riegelii* | 1 | *Enterococcus faecium* | 1 |
| *Escherichia coli* | 3 | *Klebsiella pneumoniae* | 1 |
| *Enterococcus faecalis* | 1 | *Pseudomonas aeruginosa* | 1 |
| *Klebsiella pneumoniae* | 2 | *Pseudomonas fluorescens* | 1 |
| *Lactobacillus jensenii* | 2 | *Proteus mirabilis* | 1 |
| *Micrococcus luteus* | 3 | *Staphylococcus aureus* | 2 |
| *Staphylococcus aureus* | 3 |  |  |
| *Streptococcus anginosus* | 1 |  |  |
