# Supplementary material for: The Good and the Bad: Ecological Interaction Measurements Between the Urinary Microbiota and Uropathogens
Source: Front Microbiol. 2021 May 10;12:659450. doi: 10.3389/fmicb.2021.659450 (PMC8141646; doi:10.3389/fmicb.2021.659450)
Supplement: Supplementary file 2 [file Data_Sheet_2.docx]

**Figure S1. Correlation between growth parameters.** **(A)** Correlation between doubling time and population size interactions. **(B)** Correlation between doubling time and lag phase interactions. **(C)** Correlation between the population size and lag phase interactions. There was no correlation between any of these three growth parameters, showing that these parameters are generally affected independently.

**Figure S2. Pairwise interaction matrices of commensals versus uropathogens**. Isolates with a star (*) were tested, but removed from further analyses in this manuscript due to large differences in data between triplicates and technical replicates (>10%). Figure S3 shows the observed variation in growth curves. Bacteria are listed based on their phylogeny determined by their 16S rRNA gene sequence. **(A)** Population size interactions. The response of commensal acceptors (columns) to uropathogen donors (rows). **(B)** Population size interactions. The response of uropathogen acceptors (rows) to commensal donors (columns). **(C)** Growth rate interactions. The response of commensal acceptors (columns) to uropathogen donors (rows). **(D)** Growth rate interactions. The response of uropathogen acceptors (rows) to commensal donors (columns). **(E)** Lag phase interactions. The response of commensal acceptors (columns) to uropathogen donors (rows). **(F)** Lag phase interactions. The response of uropathogen acceptors (rows) to commensal donors (columns). Generally, different isolates from the same species exhibited similar interactions.

**Figure S3. Examples of growth curves in triplicates in conditioned medium (blue) versus a control in unconditioned medium (red). (A)** Commensal *K. pneumoniae* acceptor cultured in conditioned medium of the uropathogenic donor *E*. *coli.* OD600 values of the triplicates in both conditioned medium and unconditioned medium were within 10% of each other and would be considered dependable for further analysis. **(B)** Technical replicate of the same donor/acceptor combination as in (A), a commensal *K. pneumonia* acceptor grown in the conditioned medium of an uropathogenic *E. coli*. Results from (A) and (B) were obtained months apart, showing very reproducible results (OD600 values within 10%). **(C)** Uropathogenic *E. coli* acceptor cultured in the conditioned medium of the commensal donor *L. jensenii*. Very commonly, triplicates in unconditioned medium were reproducible (OD600 value within 10% from each other), whilst results in conditioned were further apart. Results as shown here, would not be used for further analysis. Donors causing large differences in growth values are marked with (*) in figure S2. **(D)** Technical replicate of the same donor/acceptor combination as in (C). Again, in conditioned medium, the OD600 values were too dispersed to be considered for further analysis according to our stringent threshold. Between technical replicates (C) and (D) similar patterns within the triplicates were observed, showing some consistency within the heterogeneity of the responses. Note that even though there is a larger difference between the curves, the means are very similar (OD600=0.46 and 0.41 at endpoint).

**Figure S4. Correlation of 16S rDNA substitutions between donor and acceptor versus the interaction value (ε).** Gram-positive uropathogen interactions with Gram-positive commensals are more diverse than the interactions of Gram-negative uropathogens with Gram-negative commensals. This was correlated with the smaller phylogenetic distance (here expressed as the 16S rRNA gene substitutions of the 100th bp to 1400th bp region) between the Gram-negative bacteria and the Gram-positive bacteria. This is observed for all measured growth interactions (Figure 3); **(A)** Population size, **(B)** Doubling time, and **(C)** Lag phase. Mixed results show interactions of Gram-positive uropathogen donors with Gram-negative commensal acceptors and vice versa. The interaction strength was correlated with the phylogenetic distance of the interacting bacteria (p=0.041, p=0.018, and p=0.038 for population size, doubling time, and length of lag phase respectively). Significance of correlation of interaction strength and 16S substitutions was tested using a chi-square test, *X^2^* (8, *N*=358).

**Figure S5. Histograms showing the interactions of uropathogens and commensals.** Uropathogens were cultured in the conditioned medium of commensals, whilst commensals were cultured in the conditioned media of the uropathogens. Interactions between -0.22 and 0.18 were considered neutral-to-weak (log(0.8)=-0.22>ε>log(1.2)=0.18). ε values below -0.22 and above 0.18 were considered negative or positive interactions respectively. **(A)** Uropathogen donors affecting the growth rate of commensals. **(B)** Commensal donors affecting the growth rate of uropathogens. Commensal bacteria showed more negative response (ε<log(0.6)=-0.51) to the conditioned media of uropathogens than the uropathogens to the conditioned media of commensals **(C)** Change in the length of the lag phase of commensals cultured in conditioned media of uropathogens. **(D)** Change in the length of the lag phase of uropathogens cultured in the conditioned medium of commensals. Commensal acceptors respond more negatively (ε<log(0.6)=-0.51) to uropathogenic donors than uropathogenic acceptors respond to commensal donors. Commensal donors also caused more positive interactions (ε>log(1.4)=0.33) than the pathogenic donors.
