# Supplementary figures and images for: The Good and the Bad: Ecological Interaction Measurements Between the Urinary Microbiota and Uropathogens
Source: Front Microbiol. 2021 May 10;12:659450. doi: 10.3389/fmicb.2021.659450 (PMC8141646; doi:10.3389/fmicb.2021.659450)

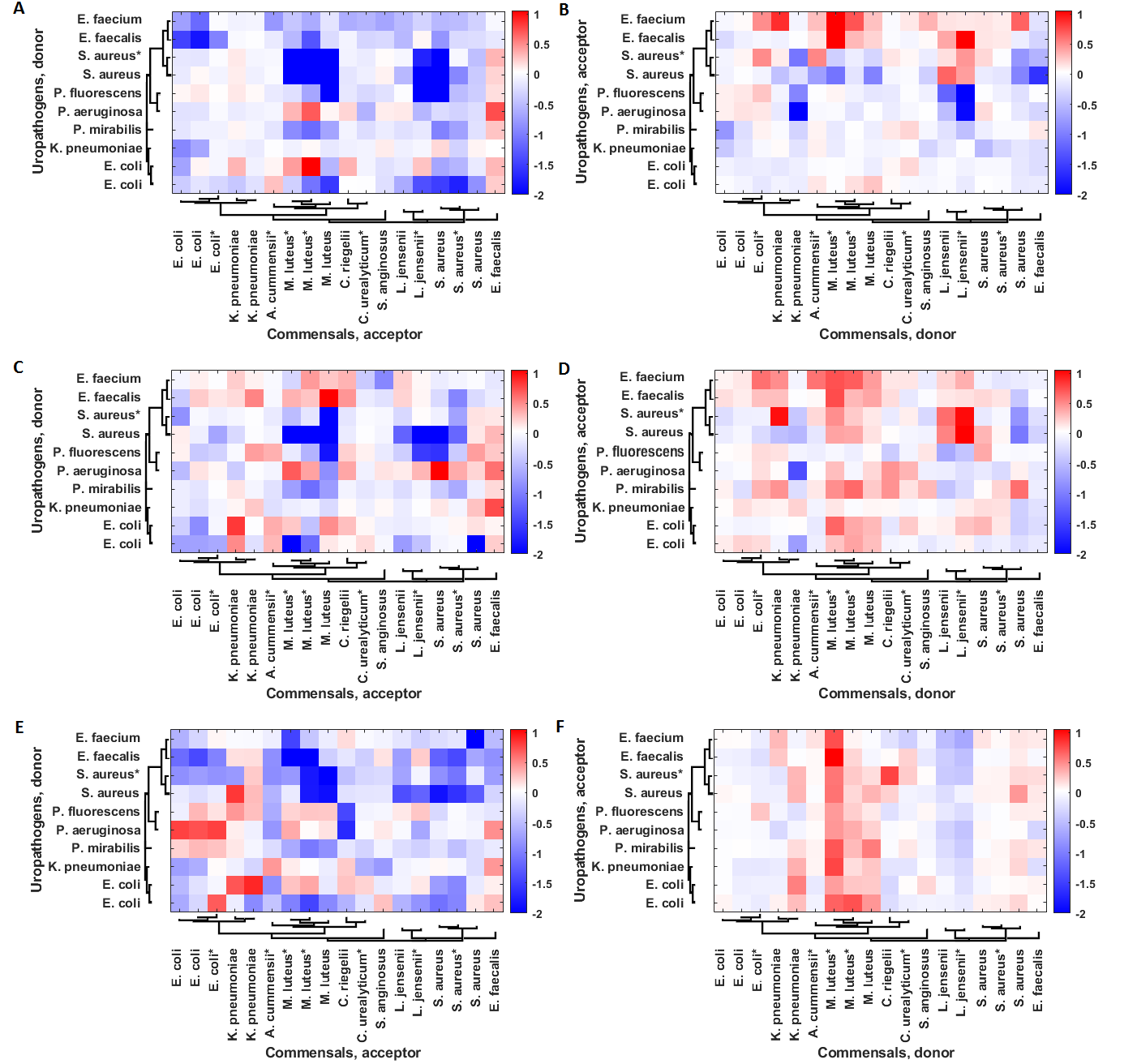

Supplement: Supplementary file 3 [file Image_1.TIF]

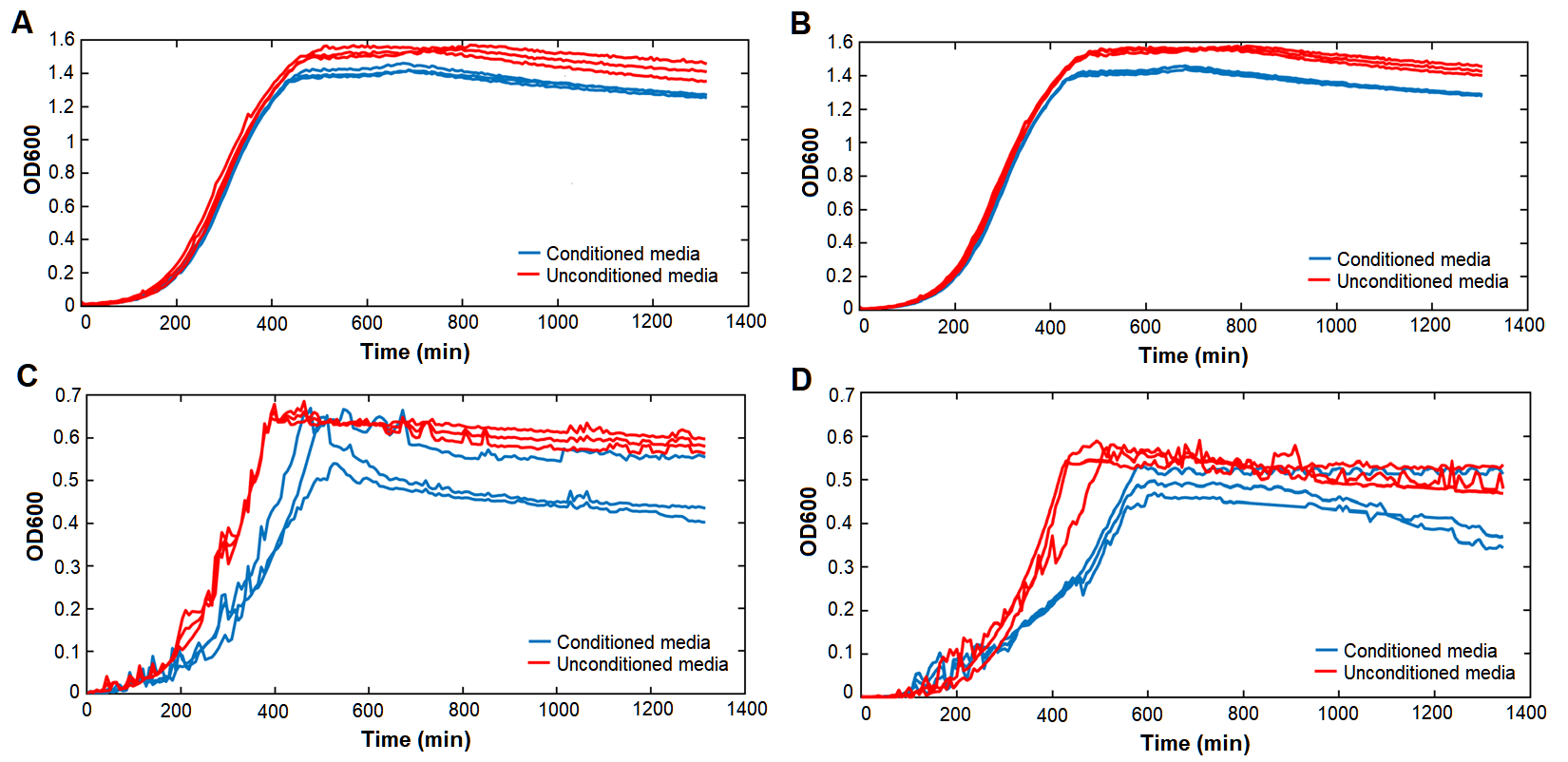

Supplement: Supplementary file 4 [file Image_2.TIF]

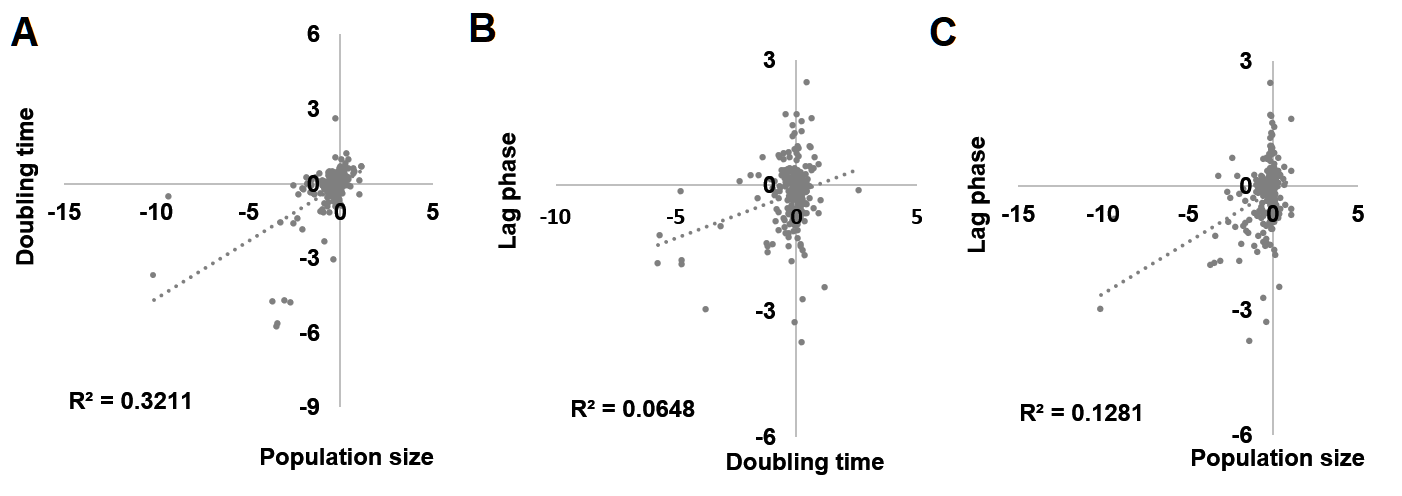

Supplement: Supplementary file 5 [file Image_3.TIF]

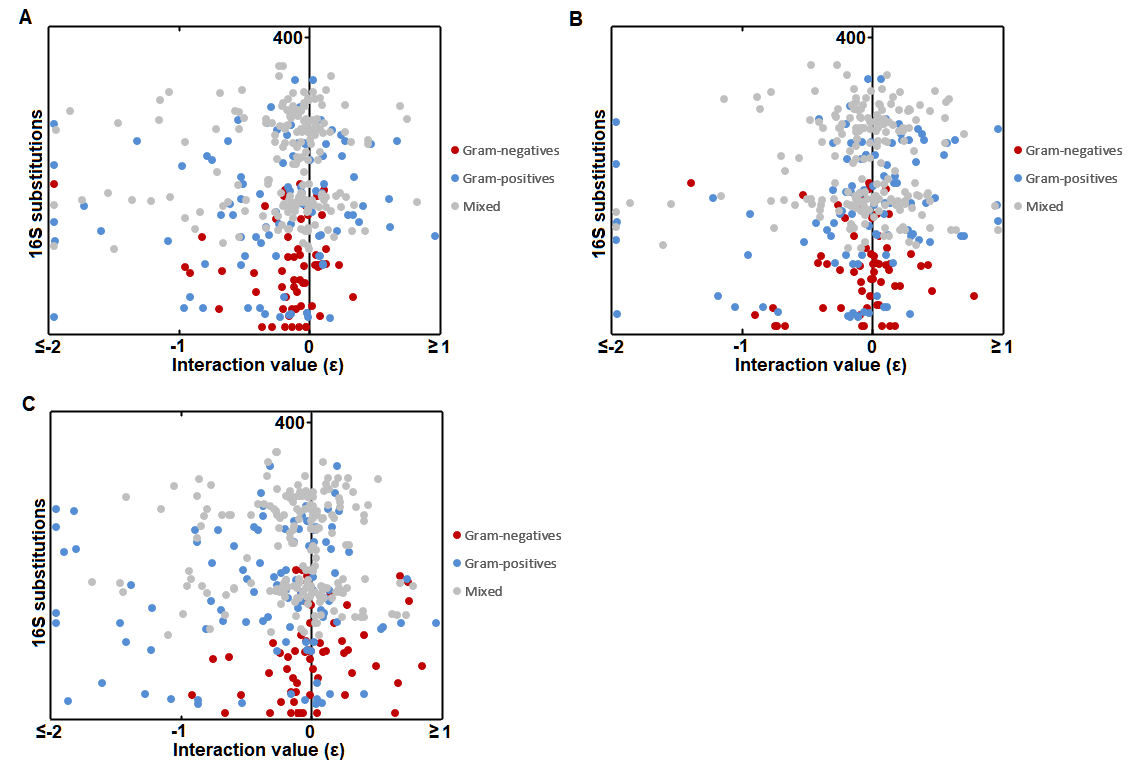

Supplement: Supplementary file 6 [file Image_4.TIFF]

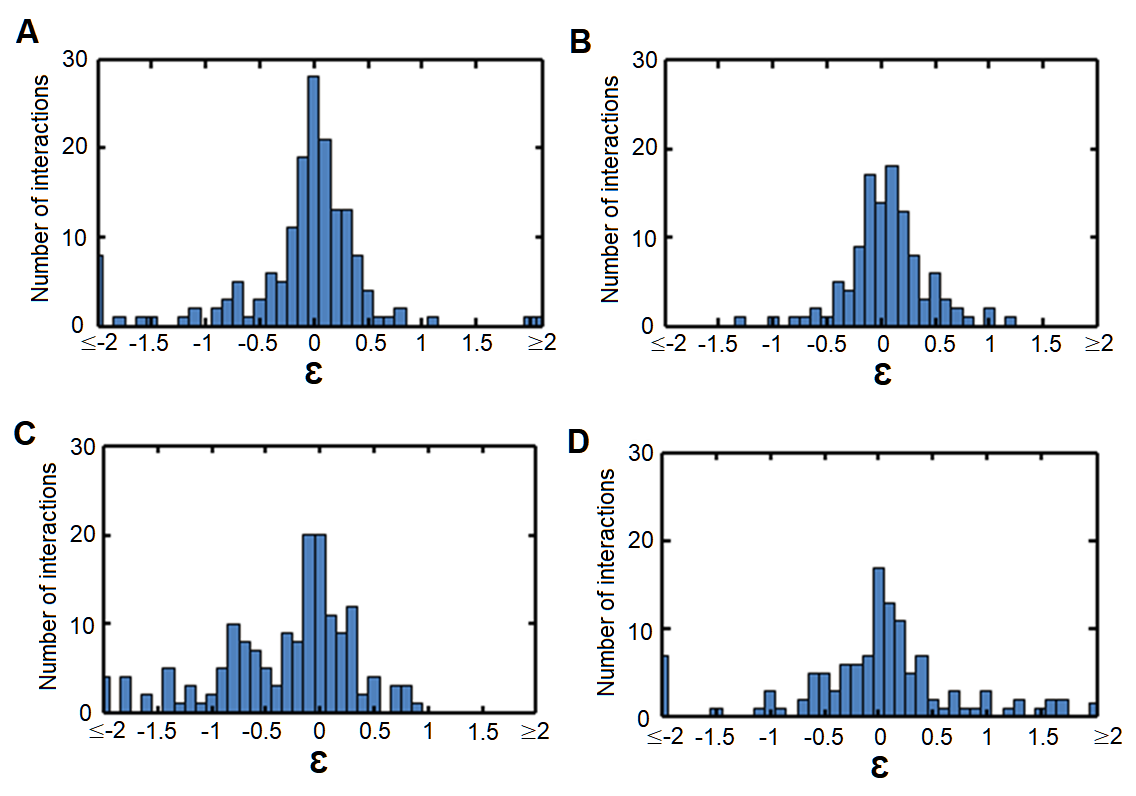

Supplement: Supplementary file 7 [file Image_5.TIFF]
